# Degradation of Mechanical Properties in HR3C Steel: The Role of σ and M_23_C_6_ Phase Evolution During Long-Term Service

**DOI:** 10.3390/nano16060344

**Published:** 2026-03-11

**Authors:** Zhun Li, Kaiyin Wang, Qianyi Zhang, Runqi Gong, Yinuo Li, Chengtai Yin, Xinying Liu

**Affiliations:** School of Mechanical and Electronic Engineering, Shandong Agricultural and Engineering University, Jinan 250100, China; sdaeu2025@126.com (Z.L.); wkywodeshijie@163.com (K.W.); 13688688063@163.com (Q.Z.); m13371009832@163.com (R.G.); 15253612891@163.com (Y.L.); 15725212185@163.com (C.Y.)

**Keywords:** HR3C steel, M_23_C_6_, microstructural, mechanical properties

## Abstract

This study systematically investigated the chemical composition, microstructure, and mechanical properties of HR3C steel tubes that have been in service. The results indicate that, after nearly 70,000 h of operation, continuous lamellar M_23_C_6_ precipitates formed along grain boundaries in the HR3C steel, with needle-like or rod-like M_23_C_6_ phases extending from the grain boundaries into the grain interiors. Additionally, NbCrN and σ-phase precipitates were observed in the regions adjacent to the grain boundaries. Mechanical testing revealed a slight increase in hardness following service exposure, while the tensile strength remained largely unchanged; the yield strength, however, increased by approximately 15%. In contrast, the elongation at fracture decreased significantly—ductility declined by 64–73% relative to the as-received condition—and impact toughness dropped dramatically by 96%. These findings collectively indicate pronounced embrittlement of the HR3C steel after long-term service at 620 °C. Microstructural analysis confirms that the precipitation of M_23_C_6_ and σ phases is the primary contributor to the observed deterioration in toughness and ductility.

## 1. Introduction

Owing to global socioeconomic advancement, nations worldwide face mounting challenges associated with environmental pollution and energy insecurity. In China, coal remains the dominant fuel for electricity generation. However, escalating fossil fuel prices—coupled with the suboptimal thermal efficiency of conventional power-generation units—have contributed substantially to environmental deterioration. As a result, both the Chinese government and power generation enterprises have prioritized the development and deployment of advanced power-generation technologies [1]. Among these, the construction of large-capacity ultra-supercritical (USC) power plants is widely regarded as a technically viable and economically efficient strategy for enhancing energy efficiency and mitigating environmental impact. To meet the stringent material requirements of USC systems, researchers have developed a range of austenitic and ferritic heat-resistant steels [2,3,4,5]. Of these, HR3C—a high-alloy austenitic stainless steel—exhibits superior high-temperature performance, including exceptional oxidation resistance and resistance to flue-gas corrosion, outperforming other 18Cr–18Ni alloys such as Super304H and TP347HG [6]. Consequently, HR3C has become one of the preferred materials for high-temperature reheaters and superheaters in USC boilers [7,8]. According to ASME SA-213, HR3C corresponds to the TP310HNbN grade: a modified 25Cr–20Ni austenitic stainless steel strengthened through controlled additions of niobium (Nb) and nitrogen (N) [9,10].

Material selection for USC components critically influences the achievable operating parameters—particularly for high-temperature sections such as superheater and reheater tubing, steam collectors, and main steam piping [11]. As key pressure-bearing components within the boiler, superheaters and reheaters must be fabricated from materials possessing adequate hardness, tensile strength, yield strength, and plastic toughness at elevated temperatures. HR3C’s high alloy content renders it susceptible to microstructural evolution—including second-phase precipitation and grain coarsening—under prolonged exposure to high temperature and stress, thereby inducing significant changes in mechanical behavior, especially impact toughness [9,12]. Accordingly, extensive research has focused on characterizing the microstructural features and mechanical response of HR3C under service-relevant conditions. Iseda et al. [9] reported that aging HR3C at 700 °C promotes M_23_C_6_ carbide formation via silicon diffusion, which subsequently triggers partial transformation into σ-phase (FeCr) and G-phase precipitates—comprising ternary intermetallic compounds such as Fe_16_Cr_6_Si_7_, Fe_16_Ni_6_Si_7_, and Fe_16_Ti_6_Si_7_. Peng et al. [13] observed sustained M_23_C_6_ precipitation along austenite grain boundaries after aging HR3C at 650 °C for 3000 h, identifying this as a primary factor driving the transition from transgranular to intergranular fracture [14]. Golanski et al. [15] identified multiple precipitate types in HR3C oxides, including M_23_C_6_ carbides, Z-phase (NbCrN), and NbX carbonitrides. Qibing et al. [16] investigated T92/HR3C heterogeneous welded joints and confirmed the presence of M_23_C_6_ carbides exhibiting dynamic recrystallization and reprecipitation behavior—contributing to enhanced tensile strength in the weld metal. Liming et al. [17] analyzed HR3C following high-temperature creep rupture at 700 °C and 750 °C, reporting NbCrN as the predominant precipitate, alongside M_23_C_6_, σ-phase, and Z-phase. Additional studies [18,19] further corroborate that Z-phase is a prevalent and potent hardening phase in HR3C; its formation significantly influences long-term strength retention. Notably, M_23_C_6_ precipitates preferentially at grain boundaries in a continuous or granular morphology, followed by progressive coarsening. In contrast, the FeCr-type σ-phase depletes chromium locally, weakens grain-boundary cohesion, and markedly reduces ductility and fracture toughness—as demonstrated by Cao et al. and supported by broader literature on austenitic heat-resistant steels [20,21,22].

After the HR3C steel pipe is used, impurities will accumulate near the grain boundaries, resulting in numerous tiny voids. In the actual operation of ultra-supercritical power plants, the stress caused by equipment vibration leads to the formation and expansion of cracks along the grain boundaries. The facts have shown that as the service time of HR3C steel pipes gradually increases, the leakage frequency has significantly risen in recent years, causing serious equipment damage and economic losses. A comprehensive characterization was conducted using optical microscopy, scanning electron microscopy (SEM), electron backscatter diffraction (EBSD), energy-dispersive X-ray spectroscopy (EDS), and standardized mechanical testing—including tensile, Charpy impact, and hardness measurements—to evaluate microstructure–property relationships and elucidate the embrittlement mechanism. The findings provide a mechanistic foundation for life assessment, failure prevention, and material optimization in next-generation USC power plants.

## 2. Materials and Methods

### 2.1. Materials

**Figure 1:**
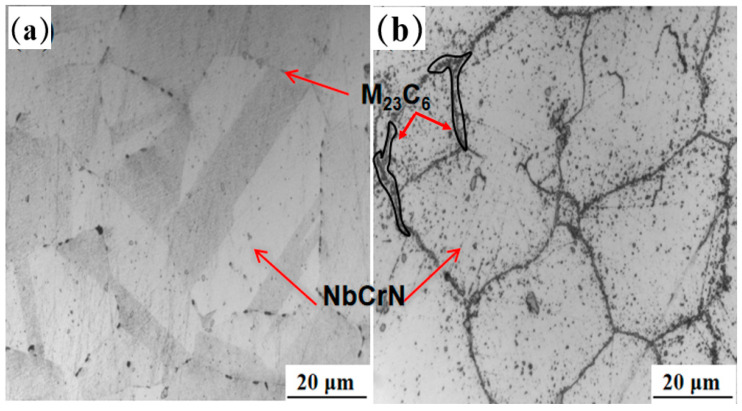
Microstructure diagram of HR3C steel, (**a**) in the received state, (**b**) after usage.

The HR3C steel samples used in this study were taken from the high-temperature outlet section of the supercritical (USC) boiler superheater tubes (Zhejiang Jiuli Hi-Tech Metals Co., Ltd., Huzhou, China). This steel pipe has passed the acceptance inspection and its dimensions are φ47.6 × 9 mm. We investigated two scenarios: (i) an in-service sample that was used for approximately 70,000 h under actual operating conditions, with a metal temperature of 623 degrees Celsius; and (ii) an original (unused) control tube as a reference benchmark. The chemical compositions of the two HR3C steel tubes are shown in Table 1, all meeting the standard requirements. Figure 1 shows the microstructure of the HR3C steel tubes, (a) presenting the typical austenitic microstructure under the receiving state, characterized by equiaxed grains and extremely low precipitation density; (b) shows the microstructure after use.

### 2.2. Experimental Methods

**Figure 2:**
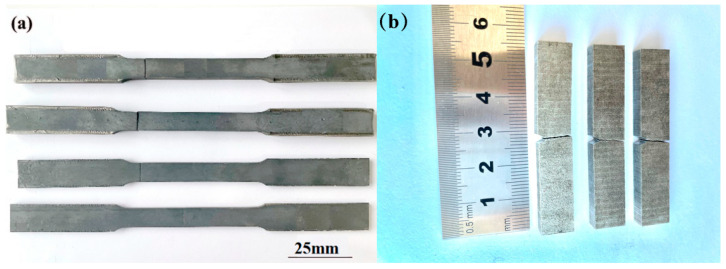
Mechanical properties specimens, (**a**) Tensile, (**b**) impact.

Vickers hardness measurements were performed on polished cross-sections using an XHBT-3000Z-III automatic Brinell hardness tester (converted to Vickers-equivalent reporting per ASTM E140) (Shanghai Shangcai Testing Machine Co., Ltd., Shanghai, China), applying a 187.5 kgf load for 10 s dwell time. Three indentations were made per specimen, and the average value was reported. For impact testing, standard 55 mm × 10 mm × 5 mm subsize Charpy V-notch specimens were machined from the tubular sections in accordance with GB/T 229–2020. Tests were conducted at room temperature (23 ± 2 °C) using a ZBC3302-A pendulum impact testing machine (MTS Industrial Systems (China) Co., Ltd., Shenzhen, China); fracture surfaces were subsequently examined via scanning electron microscopy (SEM) (CIQTEK Co., Ltd., Hefei, China). Given the limited wall thickness (<10 mm) and geometric constraints of the service-exposed tube, longitudinal arc-shaped tensile specimens were fabricated per GB/T 228.1–2021. Tensile tests were carried out at room temperature using an AG-IC Shimadzu universal testing machine (Shimadzu Corporation, Kyoto, Japan) equipped with a 100 kN load cell and extensometer for precise strain measurement. All mechanical tests employed a minimum of three replicates per condition to ensure statistical reliability. Schematic diagrams of the tensile and impact specimen geometries are shown in Figure 2.

## 3. Results and Discussion

### 3.1. Microstructure Analysis

**Figure 3:**
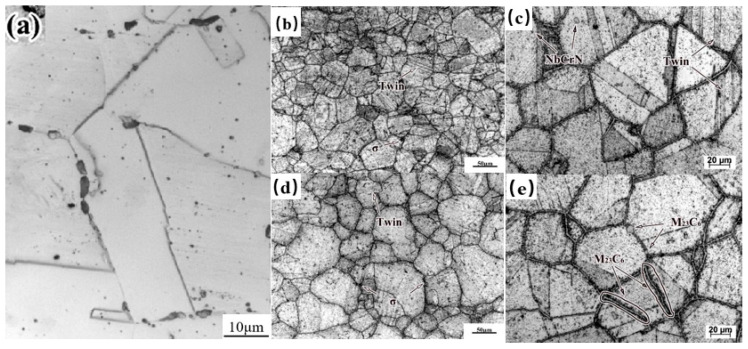
Microstructure of HR3C in As-received and service, (**a**) As-received, (**b**,**c**) fire-facing side, (**d**,**e**) the back side.

Figure 3a presents the microstructural characteristics of both the as-received and the serviced (approximately 70,000 h) HR3C heat-resistant steel tubes. The as-received tube exhibits a predominantly austenitic matrix with uniform grain size and well-defined grain boundaries, corresponding to a grain size grade of 2–3. Figure 3b–e display the microstructure of the serviced tube. Both the fire-facing side and the back side of the serviced tube retain an austenitic structure, with grain sizes ranging between grades 5 and 6. Nevertheless, a noticeable difference in austenite grain dimensions exists between the two sides. This discrepancy can be attributed to the high dislocation density accumulated during service, which stores considerable elastic strain energy and provides a driving force for recrystallization, leading to localized grain refinement [15]. Concurrently, some austenite grains undergo coalescence and growth, resulting in pronounced microstructural heterogeneity.

It has been reported that grain growth in serviced HR3C steel occurs primarily through high-angle grain boundary migration [23,24]. Due to the low stacking-fault energy of austenite, however, such migration under thermal stress can easily disrupt the atomic stacking sequence at grain boundaries [25], promoting the formation of stacking faults and annealing twins [26]. Consequently, the presence of twins in serviced HR3C steel is frequently observed. Most twins terminate within the grains as non-transgressive twins, whereas transgranular coherent twins are relatively rare.

Figure 3d,e illustrate the coarsening of austenite grain boundaries after about 70,000 h of service. According to previous studies [19], the predominant precipitates along grain boundaries under prolonged thermal exposure are M_23_C_6_ carbides, which tend to form continuous lamellar films—consistent with the observations in Figure 3e. The precipitation of M_23_C_6_ is a diffusion-controlled phase transformation, governed by both the nucleation driving force and the diffusivity of C and Cr in the austenite matrix [27]. The nucleation driving force, determined by the local concentrations of C and Cr, represents the thermodynamic propensity for M_23_C_6_ formation. Meanwhile, the diffusion rates of C and Cr atoms control their migration and ability to accumulate at preferential sites such as grain boundaries, thereby critically influencing the location and growth of carbides [28]. Given the higher energy state and faster atomic diffusion along high-angle grain boundaries, M_23_C_6_ preferentially nucleates and grows at these interfaces.

**Figure 4:**
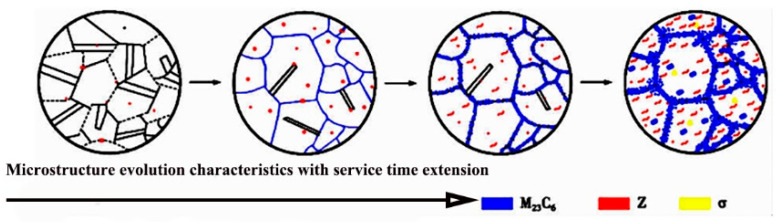
Microstructural evolution characteristics of HR3C steel with the extension of service time.

In addition to the continuous grain-boundary carbides, granular precipitates including the Z-phase (NbCrN), σ-phase (FeCr), and Nb(C, N) are also present in the serviced microstructure [9,23,26], as visible in Figure 3b. Literature suggests that at elevated temperatures, Cr diffuses into Nb(C, N) particles, gradually transforming them into a metastable NbCrN phase and eventually into the stable NbCrN phase [29]. The precipitation of NbCrN has been reported in HR3C steels at temperatures below 1200 °C [30]. This Cr- and Nb-involved phase evolution notably affects the mechanical properties, high-temperature strength, and corrosion resistance of the steel. Several researchers [22,31,32] have summarized that the microstructure and strengthening precipitates in HR3C steel undergo a three-stage evolution during long-term high-temperature service, as schematically illustrated in Figure 4.

**Figure 5:**
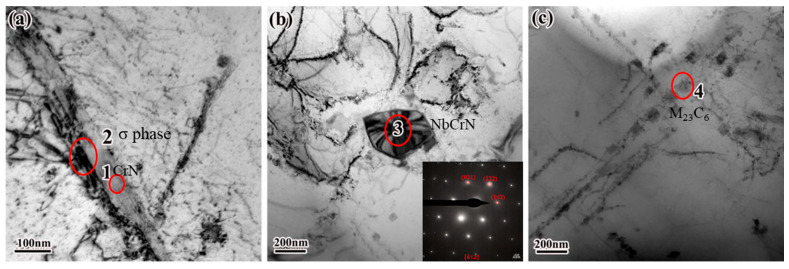
TEM test area of HR3C steel after service.

Figure 5 presents the transmission electron microscopy (TEM) images and corresponding selected-area electron diffraction (SAED) patterns of precipitates in fractured samples of service-exposed HR3C steel pipes. Four distinct regions—labeled Region 1 through Region 4—are analyzed; their elemental compositions, determined by energy-dispersive X-ray spectroscopy (EDS), are summarized in Table 2. Region 1 (Figure 5a) exhibits prominent signals of nitrogen (N), chromium (Cr), and nickel (Ni), consistent with chromium-rich nitride phases—most likely CrN. Region 2 is enriched in chromium (Cr) and iron (Fe), suggesting the presence of the σ phase (FeCr), a brittle intermetallic compound belonging to the cubic crystal system [30]. The σ phase typically forms within the temperature range of 500–900 °C and preferentially nucleates at grain boundaries—a precipitation behavior fully aligned with the operational temperature regime of HR3C steel pipes. Its formation induces localized volume expansion and chromium depletion in adjacent matrix regions, thereby promoting intergranular corrosion and degrading impact toughness. Region 3 (Figure 5b) displays strong EDS peaks for niobium (Nb), chromium (Cr), and nitrogen (N), matching the stoichiometric signature of the Z-phase (NbCrN). Region 4 (Figure 5c) reveals chain-like, branched carbide precipitates distributed along grain boundaries; notably, several elongated, parallel carbide particles exhibit an EDS-derived composition of Cr_16_Fe_5_Ni_2_C_6_—characteristic of the M_23_C_6_ type. The longest such particle exceeds 100 nm in length. Such coarse, grain-boundary-aligned M_23_C_6_ carbides significantly impair the material’s creep resistance [28].

### 3.2. Analysis of Mechanical Properties

**Figure 6:**
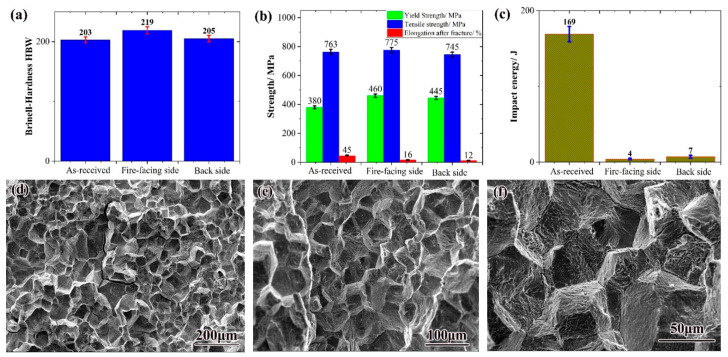
Mechanical properties and fracture morphology, (**a**) Brinell hardness, (**b**) Tensile properties, (**c**) Impact performance, (**d**–**f**) Impact Fracture Morphology.

As shown in Figure 6a, the hardness of the serviced HR3C steel shows a moderate increase on the fire-facing side compared to the initial state, while the back side remains largely comparable. The hardness distribution across the inspected tube is uniform and complies with both the ASME SA-213-2019 and GB/T 5310-2017 standards. The increase in microhardness can be attributed to two concurrent processes during high-temperature service: the precipitation of ultra-fine NbCrN particles within the austenite grains, and the recrystallization and refinement of the austenitic matrix. These mechanisms act synergistically to enhance hardness [33]. With prolonged service, the diffusion of Cr from the austenite matrix towards the grain boundaries, followed by the precipitation and growth of M_23_C_6_ carbides at these sites, further contributes to the overall hardening of the steel.

Tensile properties directly reflect the material’s structural integrity. Figure 6b presents the room-temperature tensile performance of HR3C steel in both the as-received and serviced conditions. The as-received tube exhibits a tensile strength of approximately 763 MPa and a yield strength of about 380 MPa, indicating excellent initial properties. This finding is consistent with prior studies which report that HR3C steel maintains high tensile and yield strengths even after long-term high-temperature exposure. Research by Peng et al. [34] and others has further indicated that the distribution of M_23_C_6_ carbides along grain boundaries is a primary factor influencing the tensile behavior of HR3C steel. Notably, the fracture elongation of the serviced tube is significantly lower than that of the as-received sample and falls well below the ASME standard specification. This marked reduction in ductility signifies a pronounced embrittlement tendency induced by long-term high-temperature operation.

The impact toughness of HR3C steel was also evaluated for both conditions. The as-received tube exhibited an impact absorbed energy of about 169 J, whereas the serviced tube showed a drastically reduced value of only 4–7 J, representing a decline of approximately 96% after 70,000 service hours. Such a severe loss in impact toughness critically compromises the structural safety of the alloy, particularly during operational transients such as plant start-up and shutdown, or in the event of accidental mechanical impact, where it could lead to pipe failure and jeopardize power plant safety. As concluded by Peng et al. [13], the precipitation of M_23_C_6_ at grain boundaries creates a hardness differential where the intragranular regions become harder than the boundaries themselves. This weakens the grain boundary cohesion, leading directly to the observed deterioration in impact toughness.

The fracture morphology of the impacted serviced specimens was examined, as shown in Figure 6d–f. The fracture surface appears bright and granular, exhibiting numerous reflective facets. The microscopic features reveal a characteristic “rock candy” appearance with distinct grain boundary facets, confirming that the fracture mode is predominantly intergranular brittle fracture. This observation aligns with the findings of Bai et al. [14], who studied the impact fracture mechanism of serviced HR3C steel. They reported that after exposure at 650 °C, carbide precipitation along grain boundaries leads to intergranular fracture upon impact after long-term aging. Their work further identified that the transition from a transgranular to an intergranular fracture mechanism is driven by the continuous precipitation of carbides, a process found to occur after approximately 3000 h of aging in HR3C steel.

### 3.3. Analysis of the Embrittlement Mechanism

**Figure 7:**
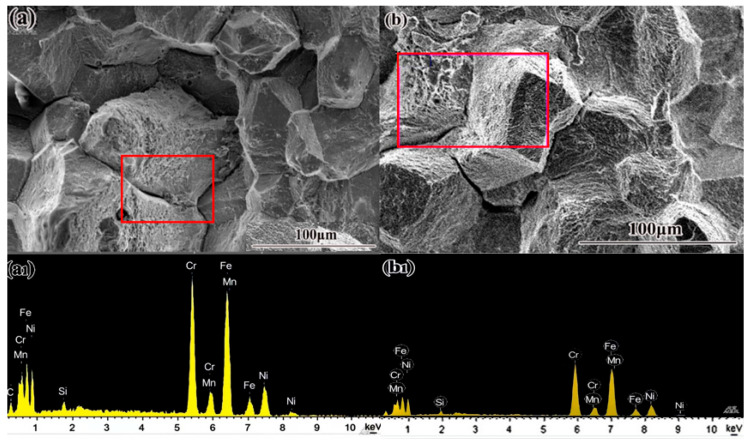
HR3C steel impact fracture morphology and energy spectrum, (**a**,**b**) Fracture morphology and energy spectrum position, (**a1**,**b1**) Energy spectrum results.

The results presented above demonstrate a clear embrittlement of HR3C steel following long-term service. Impact fracture surfaces of the embrittled samples exhibit intergranular cracking, as shown in Figure 7a,b. This observation is consistent with prior studies, which report that both impact and room-temperature tensile fractures in high-temperature-aged HR3C steel pipes are characterized by typical intergranular features [19].

Research on the as-received HR3C steel by R. Wang et al. [35] provides a baseline for comparison. In its initial state, the fracture surface displays notable microvoid coalescence in the crack extension zone, with paired minor dimples at their bottoms. Fracture in this condition involves quasi-cleavage along twin boundaries and tearing of austenitic grain boundaries. The high impact toughness of the as-received steel is attributed to strong austenite grain boundary cohesion, which forces crack propagation primarily through the grains rather than along their boundaries.

**Figure 8:**
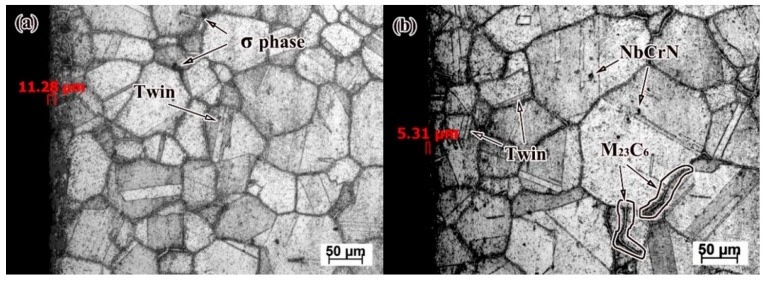
HR3C steel inner wall microstructure, (**a**) fire-facing side, (**b**) back side.

In contrast, service-exposed HR3C steel shows extensive precipitation and uniform distribution of the M_23_C_6_ phase along the grain boundaries, leading to boundary coarsening. As indicated earlier and illustrated in Figure 8a,b, the M_23_C_6_ phase grows along the boundaries into the grains, forming striated or needle-like structures with lengths of approximately 70–100 μm. This precipitation and accumulation directly cause grain boundary coarsening, which adversely affects mechanical properties by reducing toughness and increasing susceptibility to intergranular corrosion. In long-term serviced HR3C steel, the precipitation and aggregation of M_23_C_6_ at grain boundaries are therefore key factors in boundary coarsening and the associated degradation of mechanical performance.

Furthermore, the growth of Cr-rich M_23_C_6_ depletes chromium from the surrounding matrix, creating a Cr-depleted zone near the grain boundaries [36,37,38]. This depletion can lead to intergranular corrosion over extended periods. The coarsened, precipitate-filled boundaries also act as preferential sites for microcrack initiation, reducing the material’s overall deformability [13].

Concurrently, the fine-scale precipitation of M_23_C_6_ within the grains contributes to post-service hardening via precipitation strengthening, which hinders dislocation movement and crack propagation [17]. However, this benefit is offset by detrimental effects at the boundaries. The detection of Si on the impact fracture surfaces of serviced steel (Table 3 is significant, as this element promotes the formation and growth of the σ-phase at grain boundaries [17]. The presence of abundant σ-phase within the microstructure, as seen in Figure 8a, is known to induce grain boundary fragility and embrittlement [39].

Additionally, fine secondary Z-phase (NbCrN) particles, reported in fractured specimens by Zieliński et al. [40], contribute to matrix strengthening through dislocation interaction [41]. While the precipitation of these secondary phases (M_23_C_6_, σ) enhances strength, they generally lack plastic deformation capability. This characteristic, combined with their tendency to weaken austenite grain boundary cohesion, is a primary reason for the severe loss in impact toughness and the overall embrittlement observed after high-temperature service [35]. Literature further confirms that the extensive precipitation of the σ-phase during service is closely associated with the embrittlement of HR3C steel [22].

## 4. Conclusions

Based on a comprehensive investigation encompassing chemical composition, microstructure, hardness, tensile properties, impact toughness, and embrittlement mechanisms of an HR3C steel superheater tube from a 660 MW thermal power boiler, the following conclusions are drawn:

The chemical composition of the serviced HR3C steel complies with the ASTM A213/A213M-19a standard. In its initial heat-treated state, the microstructure consists of austenite with a uniform grain size (grade 2–3) and fine grain boundaries. Following long-term service, the microstructure remains austenitic, but the grain size coarsens to grade 5–6 with increased heterogeneity. The serviced material exhibits unevenly distributed granular precipitates of the NbCrN phase, σ phase, and Nb(C, N) phase. Austenite grain boundaries are coarsened and decorated with continuous granular and lamellar M_23_C_6_ carbides.

Compared to the as-received condition, the serviced HR3C steel shows increased hardness and yield strength, while tensile strength remains largely unchanged. This strengthening is attributed to the intragranular precipitation of fine NbCrN and M_23_C_6_ particles. However, a severe degradation in ductility and toughness is observed: fracture elongation decreases by 64–73%, and impact absorbed energy drops by approximately 96%, falling well below the relevant ASME standard.

The impact fracture surface exhibits a typical intergranular morphology, confirming a pronounced embrittlement tendency after prolonged high-temperature exposure. This embrittlement is primarily driven by the aggregation of the M_23_C_6_ phase along austenite grain boundaries. The subsequent precipitation of the σ phase during later stages of service further exacerbates the material’s brittleness.

## Figures and Tables

**Table 1:** Element content and heat treatment process of HR3C steel pipe.

Grade	C	Si	Mn	Cr	Ni	Nb
received	0.04	0.34	1.12	25.1	21.0	0.37
after	0.03	0.30	1.08	25.3	20.7	0.29
ASTM	0.04~0.10	≤1.00	≤2.00	24.0~26.0	19.0~22.0	0.20~0.60
heat treatment process			Solution treatment (1220 °C, 30 min)			

**Table 2:** EDS test results of HR3C steel after service.

Point	Element	Wt %	At %	Point	Element	Wt %	At %
1	N	9.12	26.36	2	/	/	/
	Si	6.89	9.92		Si	0.7	1.37
	P	0.62	0.81		/	/	/
	S	1.53	1.93		/	/	/
	V	1.22	0.97		Mn	3.4	3.4
	Cr	48.04	37.38		Cr	23.92	25.31
	Fe	4.86	3.52		Fe	51.53	50.75
	Ni	27.69	19.09		Ni	20.42	19.14
3	N	21.87	51.23	4	C	5.17	19.42
	O	5.58	11.44		S	1.39	1.95
	V	1.33	0.85		Cr	58.66	50.89
	Cr	33.96	21.42		Mn	2.73	2.24
	Fe	7.0	4.11		Fe	22.01	17.77
	Ni	1.19	0.66		Ni	10.02	7.7
	Nb	29.04	10.25		/	/	/

**Table 3:** HR3C service state impact fracture energy spectrum results.

Element	7 (a)		7 (b)	
	Weight (%)	Atomic (%)	Weight (%)	Atomic (%)
C	8.03	28.31		
Si	1.15	1.73	1.29	2.50
Cr	29.50	24.02	31.88	33.29
Mn	1.94	1.49	2.10	2.07
Fe	43.74	33.16	47.71	46.39
Ni	15.65	11.29	17.02	15.75
Total	100.00	100.00	100.00	100.00

## Data Availability

The original contributions presented in this study are included in the article. Further inquiries can be directed to the corresponding author.

## References

[1] Beér J.M. (2007). High-efficiency electric power generation: The environmental role. Prog. Energy Combust. Sci..

[2] Ning Z., Zhou Q., Liu Z., Li N., Luo Q., Wen D. (2021). Effects of imposed stresses on high-temperature corrosion behavior of T91. Corros. Sci..

[3] Yoshino M., Mishima Y., Toda Y. (2005). Phase equilibrium between austenite and mx carbonitride in a 9cr-1 mo-v-nb steel. ISIJ Int..

[4] Jie Z., Zheng H., Guo Z., Zhen Z., Zi G. (2022). Creep damage characteristics and evolution of HR3C austenitic steel during long-term creep. Mat. Sci. Eng. A.

[5] Mogire E.O., Higginson R.L., Fry A.T., Thomson R.C. (2011). Microstructural characterization of oxide scales formed on steels P91 and P92. Mater. High Temp..

[6] Bai J.M., Yuan Y., Zhang P., Zhu C.Z., Yan J.B., You C.Y. (2019). Effect of Co on the microstructure evolution of modified HR3C austenitic heat-resistant steels during long-term aging. Mater. High Temp..

[7] Wu H., Wang S., Zhao Q.X., Liang Z.Y. (2023). High-temperature corrosion data and mechanisms for T122, Super304H, and HR3C after 15 years in 1000MW ultra-supercritical power plant. Mater. High Temp..

[8] Komai N., Minami Y., Prager M., Mimura H., Igarashi M., Masuyama F., Boyles P.R. Field test results of newly developed austenitic steels in the Eddystone unit no.1 boiler. Proceedings of the ASME Pressure Vessels and Piping Conference.

[9] Iseda A., Okada H., Semba H., Igarashi M. (2007). Long-term creep properties and microstructure of SUPER304H, TP347HFG and HR3C for A-USC boilers. Energy Mater..

[10] Li J., Ma H., Wang Y. (2019). Investigation on Oxidation Behavior of Super304H and HR3C Steel in High Temperature Steam from a 1000 MW Ultra-Supercritical Coal-Fired Boiler. Energies.

[11] Wang J.Z., Liu Z.D., Bao H.S., Cheng S.C., Wang B. (2013). Effect of Ageing at 700 °C on Microstructure and Mechanical Properties of S31042 Heat Resistant Steel. J. Iron Steel Res. Int..

[12] Zhu C.Z., Yuan Y., Bai J.M., Zhang P., Yan J.B., You C.Y., Gu Y.F. (2019). Impact toughness of a modified HR3C austenitic steel after long-term thermal exposure at 650 degrees C. Mat. Sci. Eng. A-Struct..

[13] Peng B., Zhang H., Hong J., Gao J., Wang Q., Zhang H. (2010). Effect of aging on the impact toughness of 25Cr–20Ni–Nb–N steel. Mat. Sci. Eng. A.

[14] Bai X., Pan J., Chen G., Liu J., Wang J., Zhang T., Tang W. (2014). Effect of high temperature aging on microstructure and mechanical properties of HR3C heat resistant steel. Mater. Sci. Technol..

[15] Golański G., Kolan C., Zieliński A., Klimaszewska K., Kłosowicz J. (2016). Microstructure and mechanical properties of HR3C austenitic steel after service. Arch. Mater. Sci. Eng..

[16] Qi W., Rui X., Zhi W., Zhe H., Xiang J., Ju K. (2022). Microstructure and its effect on high-temperature tensile properties of T92/HR3C dissimilar weld joints. J. Manuf. Process..

[17] Xu L.M., He Y.S., Kang Y., Yeonkwan K., Jinesung J., Keesam S. (2022). Precipitation Evolution in the Austenitic Heat-Resistant Steel HR3C upon Creep at 700 °C and 750 °C. Materials.

[18] Golański G., Zieliński A., Sroka M., Słania J. (2020). The Effect of Service on Microstructure and Mechanical Properties of HR3C Heat-Resistant Austenitic Stainless Steel. Materials.

[19] Wang B., Liu Z., Cheng S.C., Liu C.M., Wang J.Z. (2014). Microstructure Evolution and Mechanical Properties of HR3C Steel during Long-term Aging at High Temperature. J. Iron Steel Res. Int..

[20] Lee J., Kim I., Kimura A. (2003). Application of Small Punch Test to Evaluate Sigma-Phase Embrittlement of Pressure Vessel Cladding Material. J. Nucl. Sci. Technol..

[21] Ji Y.S., Park J., Lee S.Y., Kim J.W., Lee S.M., Nam J., Hwang B., Suh J.Y., Shim J.H. (2017). Long-term evolution of σ phase in 304H austenitic stainless steel: Experimental and computational investigation. Mater. Charact..

[22] Cao T.S., Cheng C.Q., Zhao J., Wang H. (2019). Precipitation Behavior of σ Phase in Ultra-Supercritical Boiler Applied HR3C Heat-Resistant Steel. Acta Metal. Sin.-Engl..

[23] Nygren S., Johansson S. (1990). Recrystallization and grain growth phenomena in polycrystalline Si/CoSi_2_ thinfilm couples. J. Appl. Phys..

[24] Burke J.E., Turnbull D. (1952). Recrystallization and grain growth. Prog. Metal Phys..

[25] Gubernatorov V.V., Vladimirov L.R., Sycheva T.S., Pyatygin A.I. (2006). On the structure formation in polycrystalline metals and alloys. I: Thermocycling. Phys. Met. Metallogr..

[26] Zasimchuk I.K., Kokorin V.V., Martynov V.V. (1990). Crystal structure of martensite in heusler alloy Ni_2_MnGa. Phys. Met. Metallogr..

[27] Hong H.U., Rho B.S., Nam S.W. (2001). Correlation of the M_23_C_6_ precipitation morphology with grain boundary characteristics in austenitic stainless steel. Mater. Sci. Eng. A.

[28] Zielinski A., Golanski G., Sroka M. (2020). Evolution of the microstructure and mechanical properties of HR3C austenitic stainless steel after aging for up to 30,000 h at 650–750 degrees C. Mat. Sci. Eng. A-Struct..

[29] Zhou Y.H., Li Y.M., Liu Y.C., Guo Q.Y., Liu C.X. (2015). Precipitation behavior of type 347H heat-resistant austenitic steel during long-term high-temperature aging. J. Mater. Res..

[30] Lee K.H., Hong S.M., Shim J.H., Suh J.Y., Huh J.Y., Jung W.S. (2015). Effect of Nb addition on Z-phase formation and creep strength in high-Cr martensitic heat-resistant steels. Mater. Charact..

[31] Yang Y.H., Zhu L.H., Wang Q.J., Zhu C.C. (2014). Microstructural evolution and the effect on hardness and plasticity of S31042 heat-resistant steel during creep. Mat. Sci. Eng. A-Struct..

[32] Guan K.S., Xu X.D., Zhang Y.Y., Wang Z.W. (2005). Cracks and precipitate phases in 321 stainless steel weld of flue gas pipe. Eng. Fail. Anal..

[33] Zieliński A., Dobrzański J., Purzyńska H., Golański G. (2015). Properties, structure and creep resistance of austenitic steel Super 304H. Mater. Test..

[34] Peng B., Zhang H., Hong J., Gao J., Zhang H., Wang Q., Li J. (2011). The effect of M_23_C_6_ on the high-temperature tensile strength of two austenitic heat-resistant sheets of steel: 22Cr–25Ni–Mo–Nb–N and 25Cr–20Ni–Nb–N. Mater. Sci. Eng. A.

[35] Wang R., Duan M., Zhang J., Chen G., Miao C., Chen X., Li J., Tang W. (2021). Microstructure Characteristics and Their Effects on the Mechanical Properties of As-Served HR3C Heat-Resistant Steel. J. Mater. Eng. Perform..

[36] Terada M., Escriba D.M., Costa I., Materna-Morris E., Padilha A.F. (2008). Investigation on the intergranular corrosion resistance of the AISI 316L(N) stainless steel after long time creep testing at 600 degrees C. Mater. Charact..

[37] Kaneko K., Fukunaga T., Yamada K., Nakada N., Kikuchi M., Saghi Z., Barnard J.S., Midgley P.A. (2011). Formation of M23C6-type precipitates and chromium-depleted zones in austenite stainless steel. Scr. Mater..

[38] Yan J.B., Gu Y.F., Sun F., Xu Y.X., Yuan Y., Lu J.T., Yang Z., Dang Y.Y. (2016). Evolution of microstructure and mechanical properties of a 25Cr-20Ni heat resistant alloy after long-term service. Mat. Sci. Eng. A-Struct..

[39] Sourmail T., Bhadeshia H.K.D.H. (2005). Microstructural Evolution in Two Variants of NF709 at 1023 and 1073 K. Metall. Mater. Tran. A.

[40] Zielinski A., Sroka M., Hernas A., Kremzer M. (2016). The Effect of Long-Term Impact of Elevated Temperature on Changes in Microstructure and Mechanical Properties of Hr3c Steel. Arch. Metall. Mater..

[41] Hu Z.F., Zhang Z. (2019). Investigation of the effect of precipitating characteristics on the creep behavior of HR3C austenitic steel at 650 degrees C. Mat. Sci. Eng. A-Struct..

